# Supplementary figures and images for: Taxonomic Sampling and Rare Genomic Changes Overcome Long-Branch Attraction in the Phylogenetic Placement of Pseudoscorpions
Source: Mol Biol Evol. 2021 Feb 10;38(6):2446–67. doi: 10.1093/molbev/msab038 (PMC8136511; doi:10.1093/molbev/msab038)

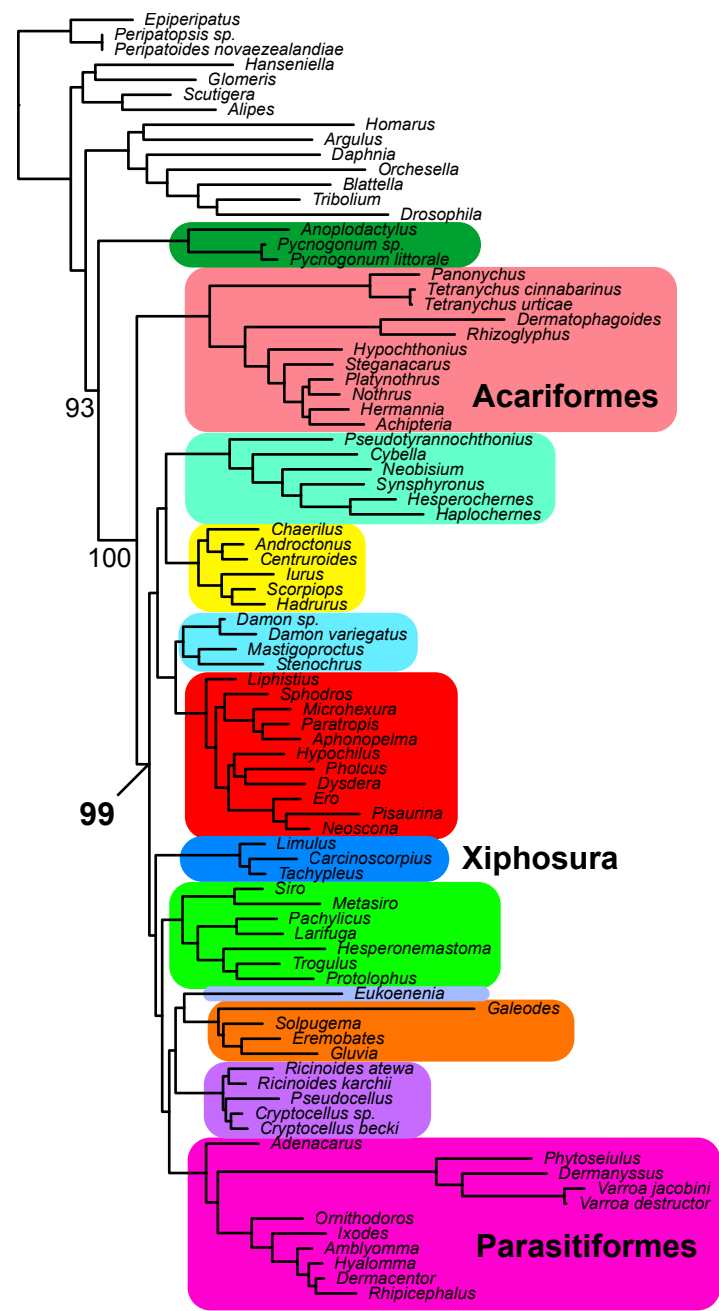

LG + F +  $\Gamma$

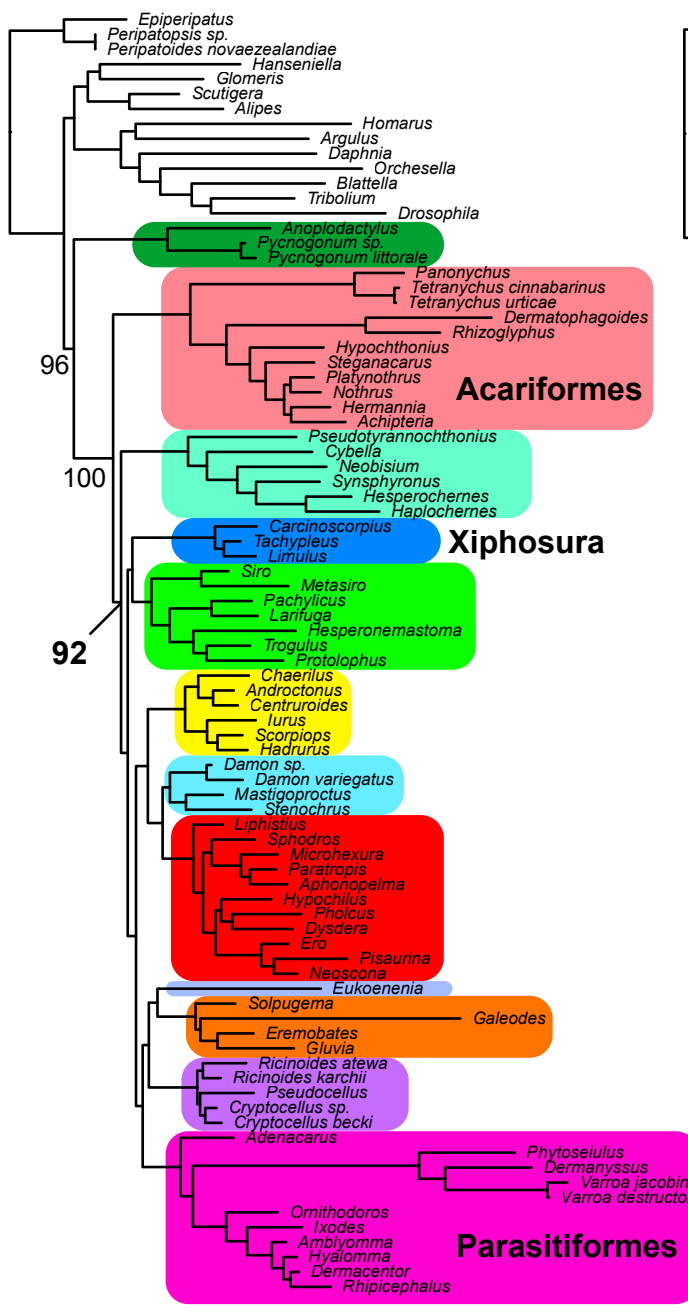

LG + R5

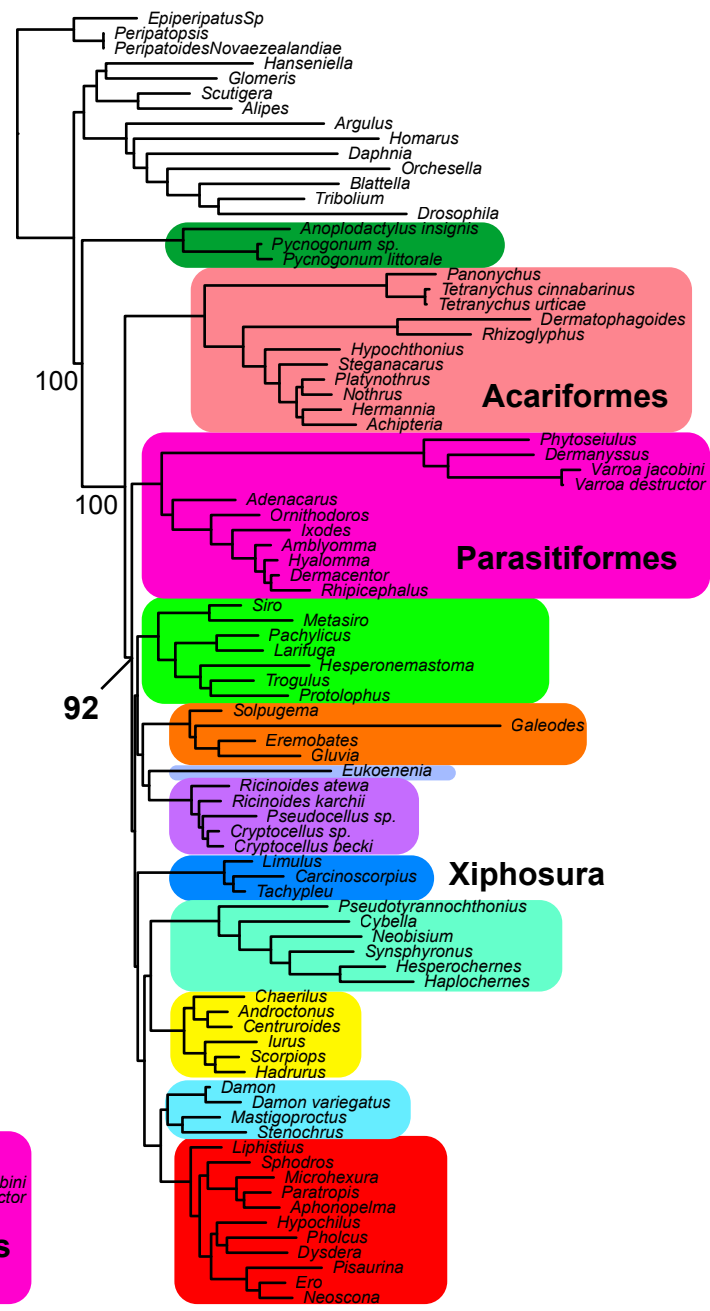

LG + C20 + R5

Supplement: msab038_Supplementary_Data [file msab038_supplementary_data.zip › Supplementary Figure S3.pdf]

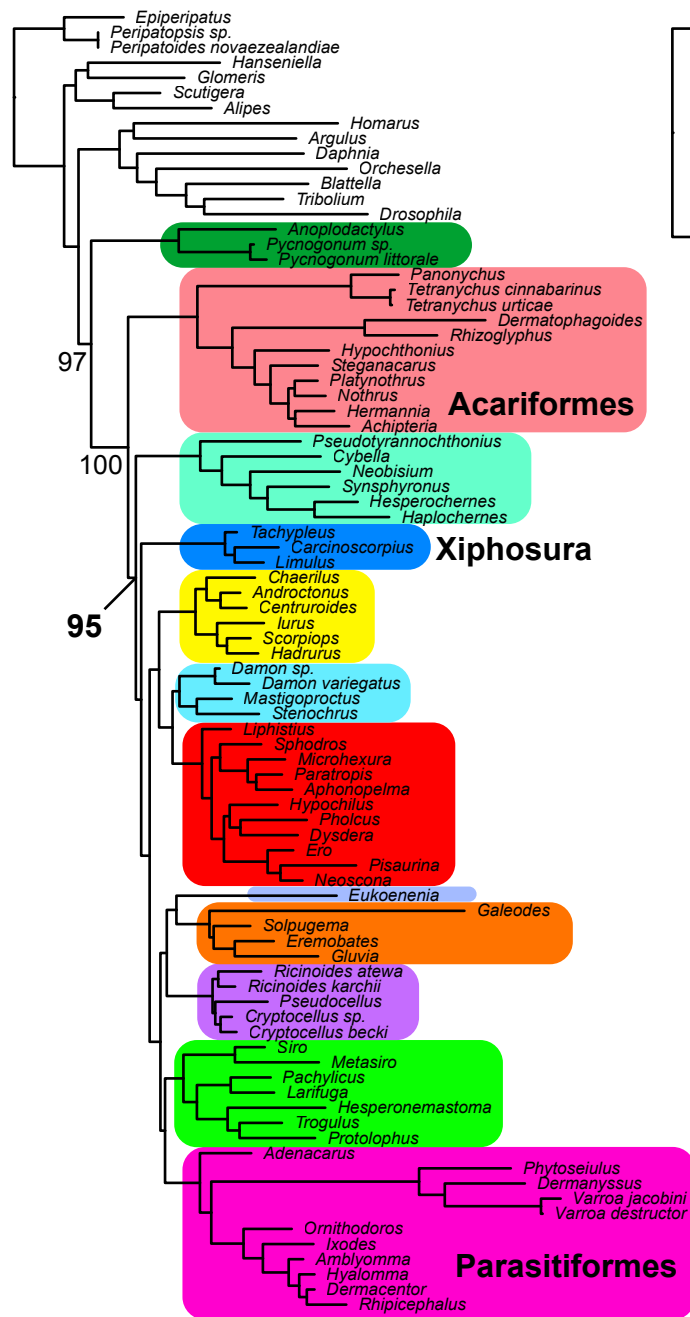

LG + F +  $\Gamma$

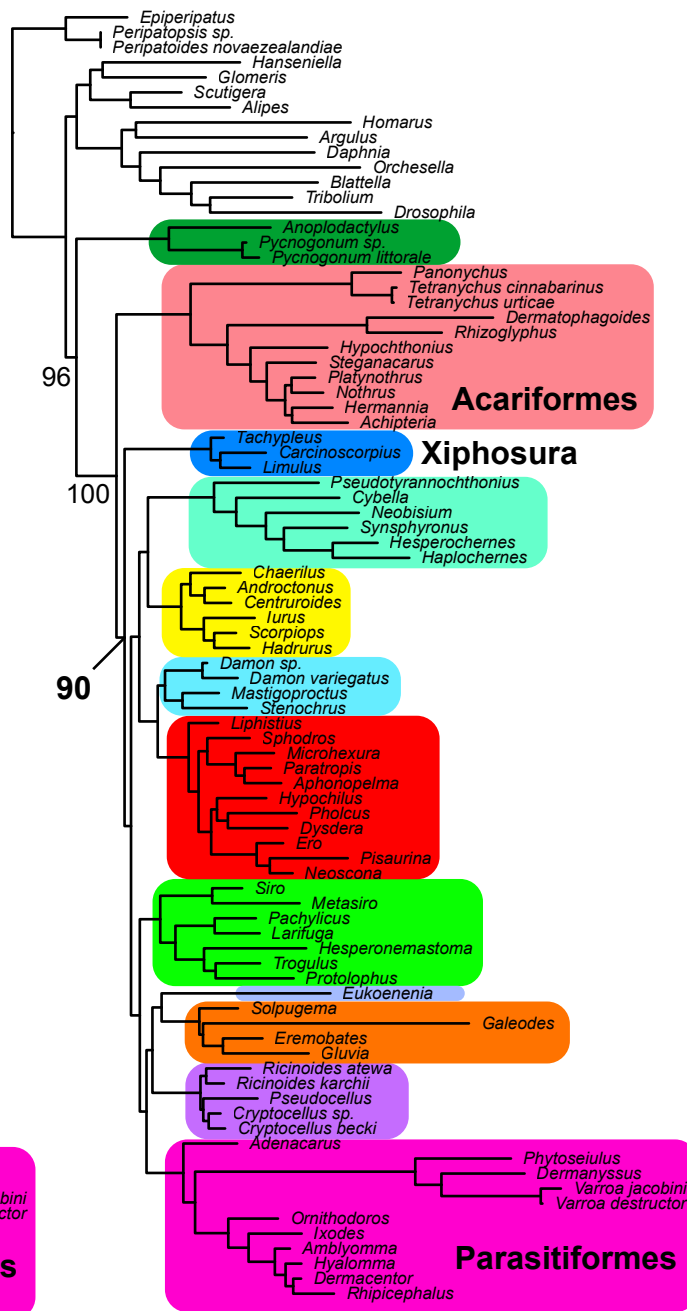

LG + R5

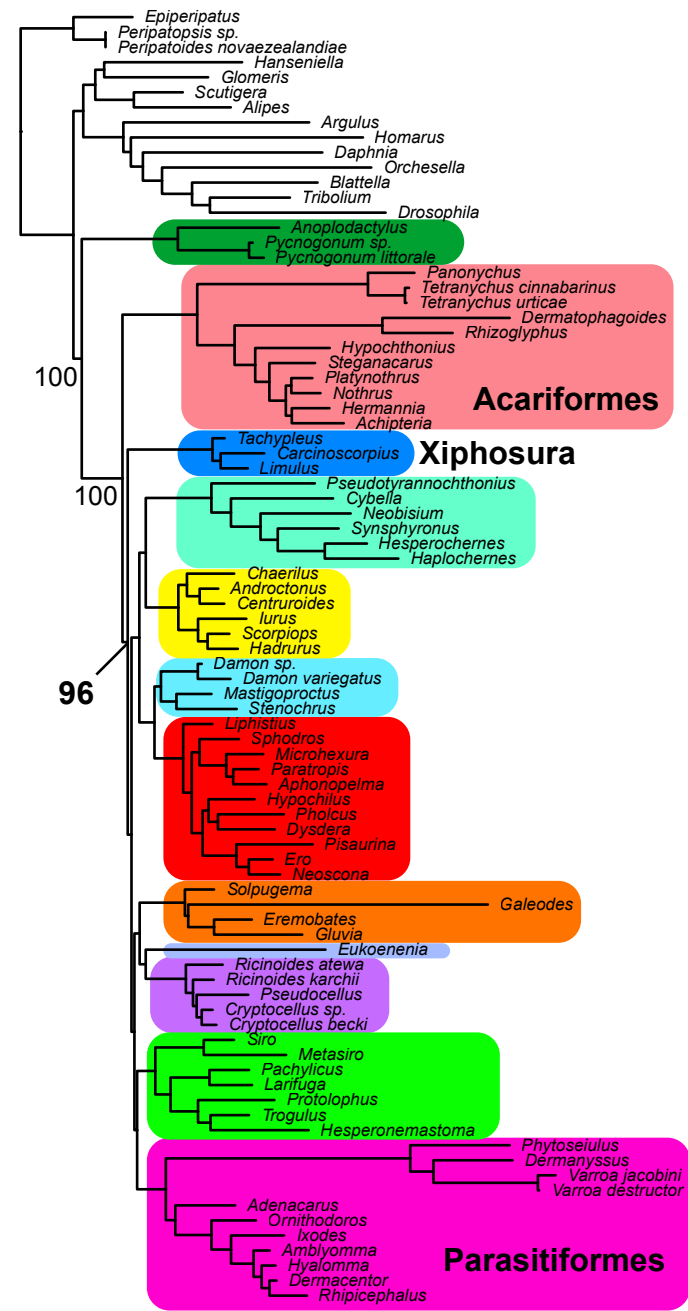

LG + C20 + R5

Supplement: msab038_Supplementary_Data [file msab038_supplementary_data.zip › Supplementary Figure S2.pdf]
