# Supplementary material for: Taxonomic Sampling and Rare Genomic Changes Overcome Long-Branch Attraction in the Phylogenetic Placement of Pseudoscorpions
Source: Mol Biol Evol. 2021 Feb 10;38(6):2446–67. doi: 10.1093/molbev/msab038 (PMC8136511; doi:10.1093/molbev/msab038)
Supplement: msab038_Supplementary_Data [file Supplementary Figure S1 - DGLS.pdf › Supplementary Figure S1 - DGLS.pdf]

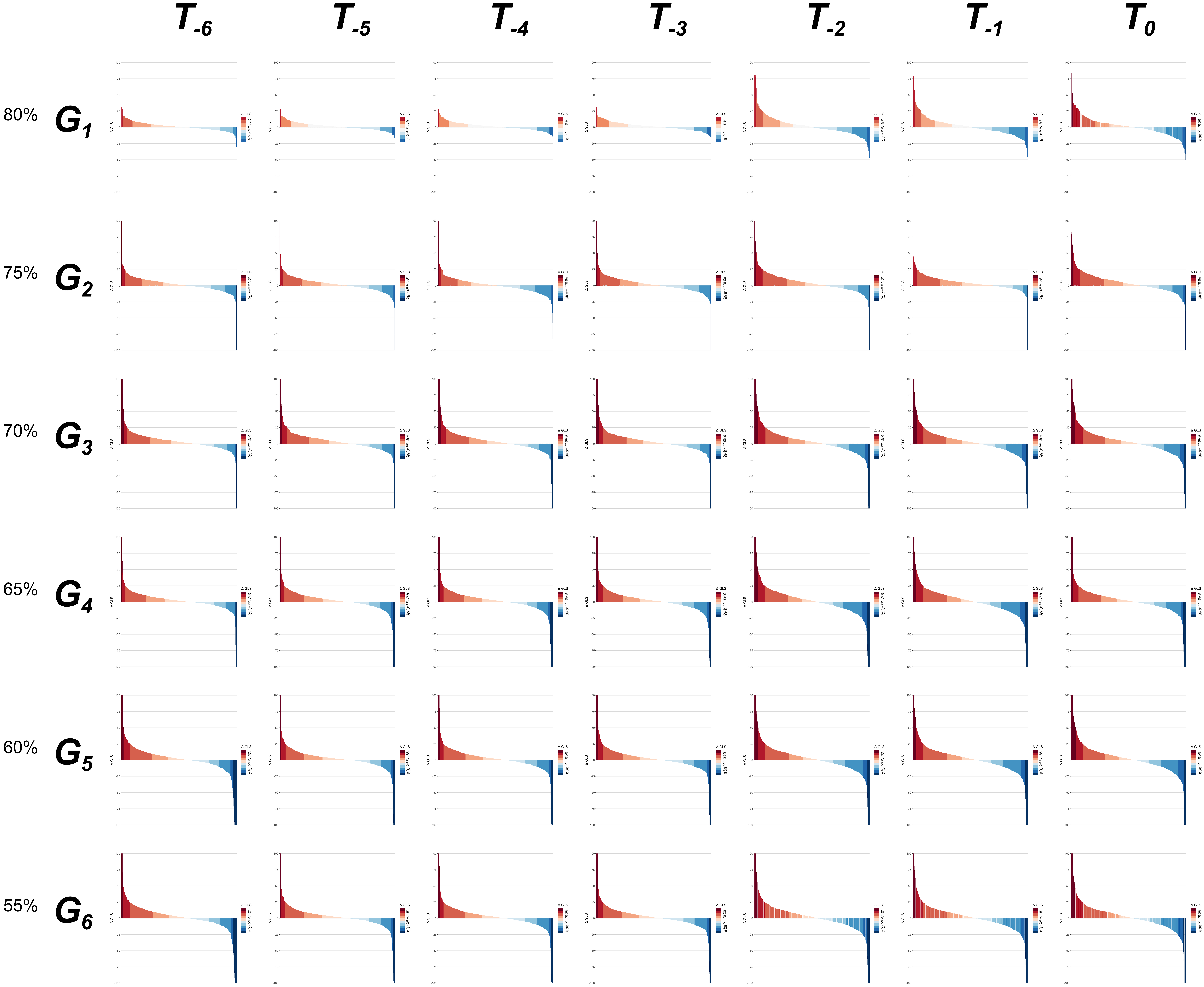

T-6
T-5
T-4
T-3
T-2
T-1
T0
80% G1
75% G2
70% G3
65% G4
60% G5
55% G6
Δ GLS
